# Supplementary material for: High temperature singlet-based magnetism from Hund’s rule correlations
Source: Nat Commun. 2019 Feb 7;10:644. doi: 10.1038/s41467-019-08497-3 (PMC6367396; doi:10.1038/s41467-019-08497-3)
Supplement: Supplementary file 1 — Supplementary Information [file 41467_2019_8497_MOESM1_ESM.pdf]

Supplementary information for

High temperature singlet-based magnetism from Hund's rule correlations

Lin Miao,[1,2] Rourav Basak,[1] Sheng Ran,[3] Yishuai Xu,[1] Erica Kotta,[1] Haowei He,[1] Jonathan D. Denlinger,[2] Yi-De Chuang,[2] Y. Zhao,[3,4] Z. Xu,[3] J. W. Lynn,[3] J. R. Jeffries,[5] S. R. Saha,[3,6] Ioannis Giannakis,[7] Pegor Aynajian,[7] Chang-Jong Kang,[8] Yilin Wang,[9] Gabriel Kotliar,[8] Nicholas P. Butch,[3,6] L. Andrew Wray,[1]*

[1] Department of Physics, New York University, New York, New York 10003, USA
[2] Advanced Light Source, Lawrence Berkeley National Laboratory, Berkeley, CA 94720, USA
[3] NIST Center for Neutron Research, National Institute of Standards and Technology, Gaithersburg, Maryland 20899, USA
[4] Department of Materials Science and Engineering, University of Maryland, College Park, Maryland 20742, USA
[5] Materials Science Division, Lawrence Livermore National Laboratory, Livermore, CA 94550
[6] Center for Nanophysics and Advanced Materials, Department of Physics, University of Maryland, College Park, Maryland 20742, USA
[7] Department of Physics, Applied Physics and Astronomy, Binghamton University, Binghamton, NY 13902
[8] Department of Physics and Astronomy, Rutgers University, Piscataway, New Jersey 08854-8019, USA
[9] Department of Condensed Matter Physics and Materials Science, Brookhaven National Laboratory, Upton, New York 11973, USA

Supplementary Note 1. Uranium f-electron charge density versus multiplet symmetry

There are at present very few multiplet-resolving measurements in the literature capable of distinguishing an effective local $5f^1$ symmetry. However, the f-electron charge density can be characterized from a wide range of deeper core levels, and very rarely shows just ~1 f-electron state within uranium compounds. When comparing between multiplet- and density-resolving spectroscopic approaches, one must bear in mind that the lowest order expectation with an electron-rich ligand (like Bi/Sb) and low-f-electron density metal (like U) is that f-electron density should have a significantly higher value than the multiplet symmetry would indicate.

This is well known from a wide range of materials, such as transition metal oxides. For example, numerical models for insulating VO_2 give roughly two 3d electrons per vanadium atom (1.83 to 2.48 d-electrons from different modeling approaches in Ref. [1]), even though the multiplet symmetry is unambiguously $3d^1$-like. Likewise, the DFT+DMFT valence histogram restricted to a single-atom basis (as in Fig. 4a of the main text) shows that the occupancy of the USb_2 U 5f orbital is 2.17, representing a nominal $5f^2$ valence with weak mixed-valence character due largely to metal ligand hybridization.

Supplementary Note 2. Exclusion of R2 XAS data due to the Fano effect

Unlike the case for X-ray measurements, the strong non-resonant photoemission that occurs in the VUV can result in significant Fano interference that greatly modifies broader TEY XAS features [2]. This amplitude of this Fano interference depends on properties of the low energy band structure that are not well captured in a multiplet model. The approximate effect of TEY Fano interference on the XAS spectrum of URu_2Si_2 was modeled in our earlier work [3], and the results have been reproduced in Supplementary Figure 1. The simulation shows that Fano interference shifts R2 on a highly problematic energy scale of ~1 eV, while the corresponding energy shift in R1 is quite small (<~0.020 eV).

One might think that measuring XAS by the total fluorescence yield (TFY) method would provide a good workaround. Unfortunately however, extreme ultraviolet TFY tends to be influenced to a similar or greater degree by the elastic photon Fano effect [4], and does not provide an effective route to overcoming the issue.

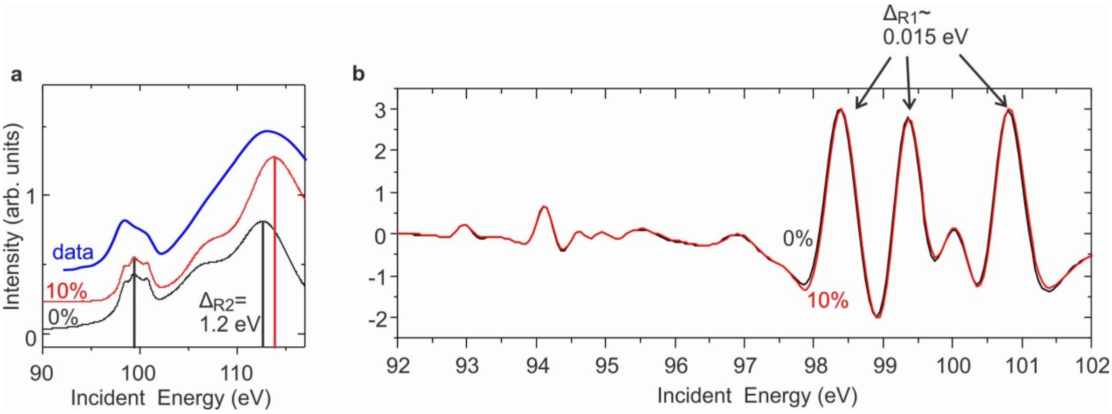

Supplementary Figure 1: Effect of Fano interference on R2. a, The O-edge XAS spectrum of (blue) an USb_2 surface is compared with (black) a standard multiplet simulation for U^{4+} and (red) the same simulation after incorporating moderate Fano interference. Vertical lines indicate the simulated R1 and R2 intensity maxima, which are offset by Δ = 1.2 eV by Fano interference at R2. **b**, SDI curves are shown for the 2 simulations, near R1. The primary R1 features are offset by just $\Delta \sim$ 0.015 eV. Panel (a) is adapted from Ref. [4].

Supplementary Note 3. The uneven surface topography of UBi_2.

The vacuum cleaved surface of USb_2 was studied by scanning tunneling microscopy (STM) in Ref. [5], revealing a clean cleave within the [001] plane, and a surface terminated by antimony atoms. No such STM work on UBi_2 has previously been reported. An STM topographic map of vacuum-cleaved UBi_2 is shown in Supplementary Figure 2, revealing an uneven surface with multiple non-parallel cleavage planes. No large flat area with clear atomic resolution was found for most parts of the sample. Only small, spatially bounded regions were observed with a square lattice structure matching the [001] facet of nominal cleavage (see Supplementary Figure 2a, inset). Rough surfaces of this sort can be highly problematic for techniques specifically sensitive to the outermost atomic layer, such as angle resolved photoemission (ARPES) and STM. The uneven cleavage also limits the quantitative analysis of total electron yield X-ray absorption spectroscopy (TEY XAS), which is expected to have a ~>2 nm probe depth (see Methods).

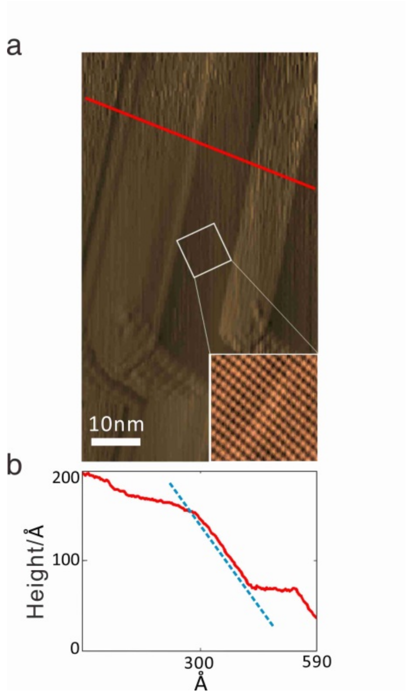

Supplementary Figure 2: The cleaved surface of UBi_2. a, A 60 *100nm STM topographic map of vacuum-cleaved UBi_2 shows multiple non-parallel surfaces. Only select regions can be identified as the [001] plane (insert). The measured in-plane lattice constant is around 0.45nm, matching the lattice constant of UBi_2 (a=0.4445 nm). **b,** The height profile along the red line in (a). The blue dashed line indicates a terrace with atomic structure matching the (001) facet.

Supplementary Note 4. The linear dichroic effect in UBi_2

The linear dichroic effect at peak-A ($h\upsilon$=99.2eV) of UBi_2 is shown in Supplementary Figure 3a. Other than at the lowest temperature (T=15K), the XLD of UBi_2 is not significantly distinguished from the systematic error bars from data normalization (~2%). The XLD amplitude is positive throughout the R1 O-edge resonance region (shaded area in Supplementary Figure 3b), and does not show temperature dependence with sufficient amplitude for a close analysis.

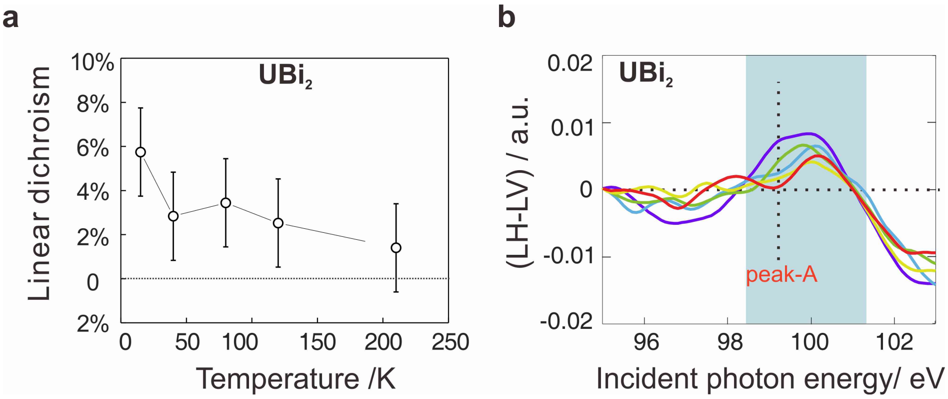

Supplementary Figure 3: The analysis of XLD of UBi_2. a, The linear dichroism of UBi_2. **b**, A shaded area (from 98.5eV to 101.4eV) where the XAS peaks of UBi_2 resident. All of this area is dominated by the positive XLD, and lack of negative XLD.

Supplementary Note 5. CEF symmetries of $5f^2$ uranium in USb_2

Hund's rule interactions for a single $5f^2$ uranium atom create a 9-fold degenerate ground state basis with angular momentum J=4. These states are further split by the crystal electric field (CEF) into five singlet states and two doublets. The singlet states include Γ_1 ($|m_J|$=0, 4 moment components), Γ_2 ($|m_J|$=4) and Γ_3 ($|m_J|$=2). The doublets are derived from $|m_J|$=1, 3 components, and include the Γ_5 state with occupancy comparable to the CEF ground state at T>~100K in the AM+MF model. These symmetry labels are identical to those of URu_2Si_2 [4, 6], and represent mixing over +/-4 units of angular momentum by a crystal field with C_4 rotational symmetry.

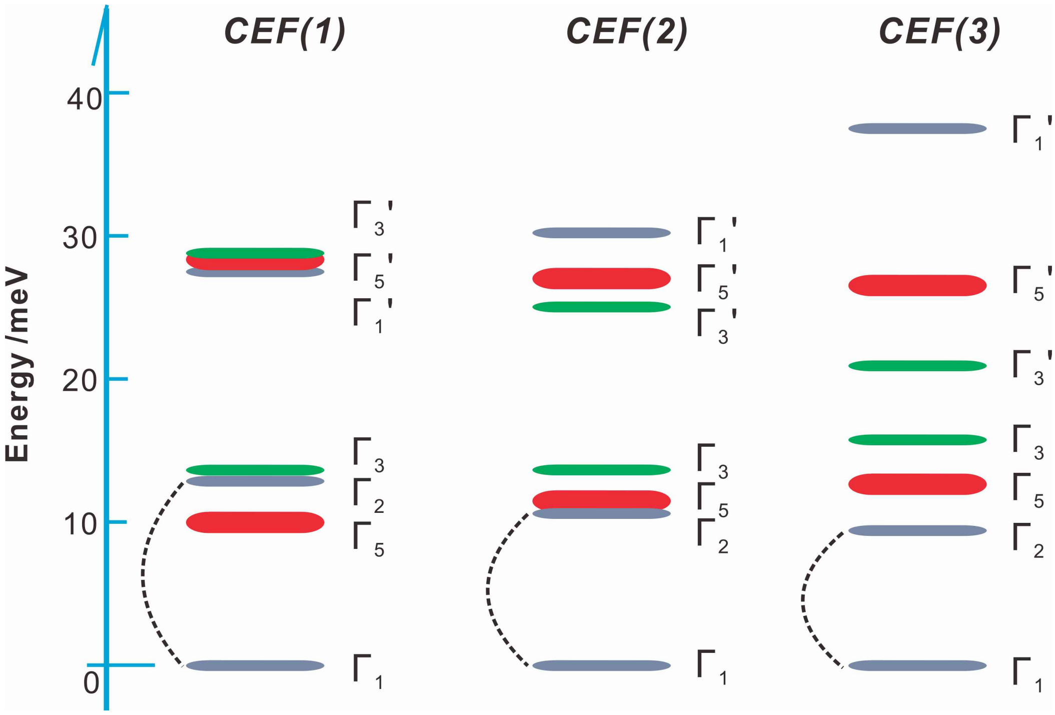

Supplementary Figure 4: The paramagnetic CEF symmetries of $5f^2$ uranium. The *CEF(1)* crystal field scheme is compared with alternatives (*CEF(2) and CEF(3)*), as defined by paramters listed in table 1 of the main text. The doublet states (Γ_5 and Γ_5^1) are plotted with thicker lines. A dashed line is drawn connecting the Γ_1 and Γ_2 singlets, which can generate z-axis magnetic moment when combined coherently. Beneath the Neel temperature, the Γ_1 state becomes mixed with Γ_2, and the Γ_5 doublet splits into magnetically polarized $\Gamma_5(1)$ and $\Gamma_5(2)$ branches

Supplementary Note 6. Oxygen L_1-edge XAS

The USb_2 and UBi_2 samples are surveyed by XAS at the oxygen L_1-edge in Supplementary Figure 5. Both samples are found to have a broad peak around 42eV, which is very similar to the oxygen L_1-edge seen from a partially oxidized iron selenide (O-FeSe) sample (Supplementary Figure 5a). Negative second derivative curves show two distinctive sub-peaks (Supplementary Figure 5b). The first peak is located at 41.6eV (peak A), and is found in all three samples. The second one is at 43eV (peak B), within ~1eV of the nominal binding energy of uranium 6s-electrons (uranium P_1-edge), and is absent for the FeSe sample. By fitting the USb_2 and UBi_2 spectra with Voigt functions at peak A and peak B, we find that the ratio of peak intensities $I(A)/I(B)$ is 15% smaller for USb_2, suggesting that there may be approximately 15% less oxygen at the USb_2 surface if the 43eV peak is attributed to uranium. This 15% value is comparable to error bars from the background subtraction and fitting procedures, and one can only conclude that the measurement is suggestive of a similar oxygen concentration at the surface of both samples.

The above analysis is complicated by the uncertainty in the attribution of peak B. It is worth noting that the absolute intensity of the ~41eV XAS signal was also comparable between the two samples, with somewhat more intensity (47% more) fitted in the UBi_2 spectrum. This corroborates the idea that there may be a higher oxygen concentration at the surface of this sample, but does nothing to improve error bars, as absolute XAS intensity tends to be unreliable when comparing between different samples.

The role of oxygen at the surface is unclear, but one can assume that some is present in the form of adsorbed polar molecules such as H_2O, CO, and CO_2, which are present in the vacuum, and will play a role in screening the polar surface presented by the UX_2 structure.

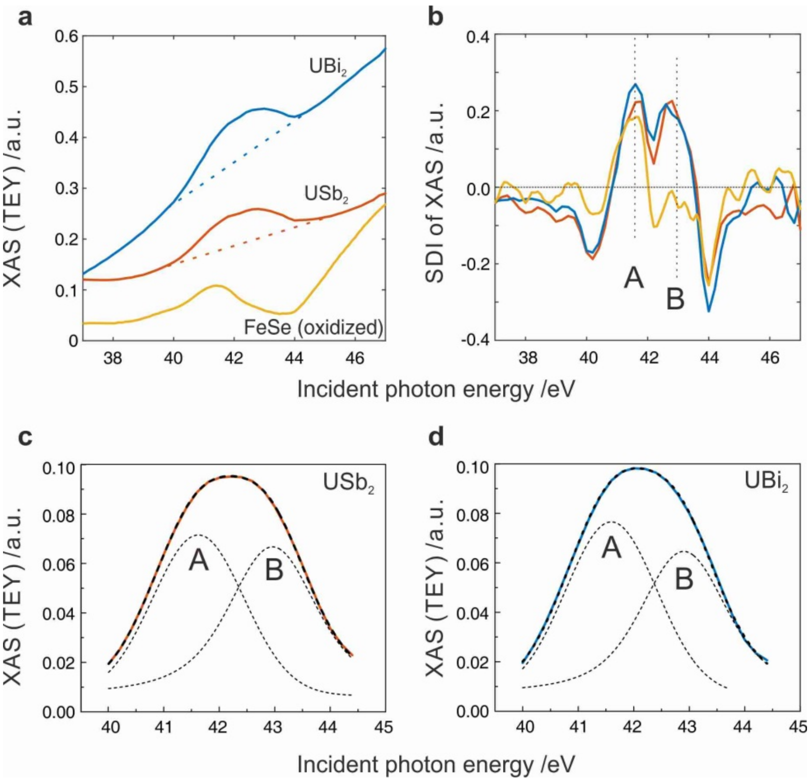

Supplementary Figure 5: Oxygen L-edge XAS. a, The XAS (TEY) spectra of UBi_2 (blue), USb_2 (red), and a partially oxidized FeSe sample (yellow). **b,** The negative second derivative curves of XAS spectra in (a) Two peaks are labeled A (41.6eV) and B (43.0eV). **c-d,** The XAS of USb_2 (c) and UBi_2 (d) are fitted with two Voigt functions after subtraction of the linear backgrounds drawn in panel (a). The raw data in panel (c) are scaled up by a relative factor of 1.5, compared with raw data underlying panel (d).

Supplementary Note 7. Hartree-Fock renormalization

In the main text, two different sets of Hartree-Fock parameters are used to simulate the XAS spectra of UBi_2 and USb_2, as described in Methods. Supplementary Figure 6 shows that the XAS simulations remain very similar if these Hartree-Fock parameter sets are reversed.

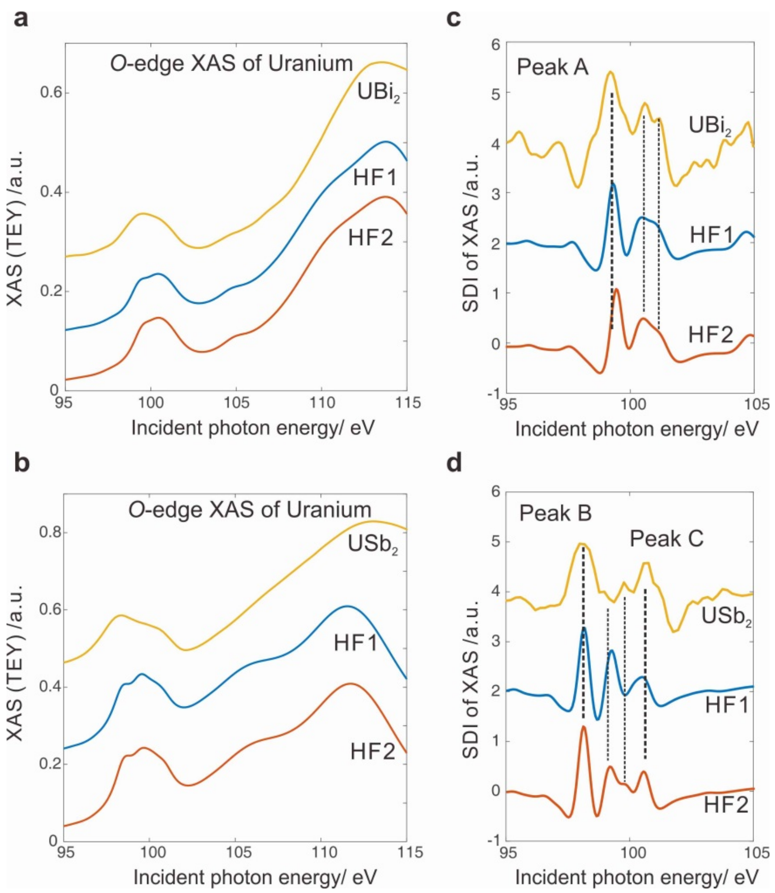

Supplementary Figure 6: **Hartree-Fock renormalization and the XAS spectrum. a-b,** The uranium O-edge XAS spectra of (a) UBi_2 and (b) USb_2 are compared with simulated XAS spectra fitted with the parameter sets used for UBi_2 (labeled HF1) and USb_2 (labeled HF2). **c-d,** The negative SDI curves extract from the experimental XAS data and simulated curves in (a) and (c) The labels Peak-A (98.2eV), Peak B (99.2eV) and Peak C (100.8eV) are used to mark the main features in the XAS spectra, as in the main text.

Supplementary References

[1] Haverkort, M. W. et al., Orbital-Assisted Metal-Insulator Transition in VO_2, *Phys. Rev. Lett.* **95**, 196404

[2] Ogasawara, H. & Kotani, A. Calculation of Magnetic Circular Dichroism of Rare-Earth Elements. *J. Phys. Soc. Jpn*. **64**, 1394 (1995).

[3] Wray, L. A. et al. Measurement of the spectral line shapes for orbital excitations in the Mott insulator CoO using high-resolution resonant inelastic x-ray scattering. *Phys. Rev. B* **88**, 035105 (2013).

[4] Wray, L. A. et al. Spectroscopic determination of the atomic f-electron symmetry underlying hidden order in URu2Si2, *Phys. Rev. Lett.* **114**, 236401 (2015).

[5] Chen, S.-P. et al. Determination of surface structure of cleaved (001) USb_2 single crystal,

Philosophical Magazine, **89**:22-24, 1881-1891 (2009).
[6] Sundermann, M. et al. Direct bulk sensitive probe of 5f symmetry in URu_2Si_2, *Proc. Natl. Acad. Sci. (USA)* **113**, 13989 (2016).
